# Interplay between Forced Convection and Electroconvection during the Overlimiting Ion Transport through Anion-Exchange Membranes: A Fourier Transform Analysis of Membrane Voltage Drops

**DOI:** 10.3390/membranes13030363

**Published:** 2023-03-21

**Authors:** Lorena Hernández-Pérez, Manuel César Martí-Calatayud, Maria Teresa Montañés, Valentín Pérez-Herranz

**Affiliations:** IEC Group, ISIRYM, Universitat Politècnica de València, Camí de Vera s/n, 46022 València, Spain

**Keywords:** electroconvection, electrodialysis, anion-exchange membranes, Fourier transform, forced convection, overlimiting currents

## Abstract

Electrodialysis (ED) applications have expanded in recent years and new modes of operation are being investigated. Operation at overlimiting currents involves the phenomenon of electroconvection, which is associated with the generation of vortices. These vortices accelerate the process of solution mixing, making it possible to increase the transport of ions across the membranes. In this work, frequency analysis is applied to investigate the interaction between different parameters on the development of electroconvection near anion-exchange membranes, which would provide a basis for the development of ED systems with favored electroconvection. Chronopotentiometric curves are registered and Fast Fourier Transform analysis is carried out to study the amplitude of the transmembrane voltage oscillations. Diverse behaviors are detected as a function of the level of forced convection and current density. The synergistic combination of forced convection and overlimiting currents leads to an increase in the signal amplitude, which is especially noticeable at frequencies around 0.1 Hz. Fast Fourier Transform analysis allows identifying, for a given system, the conditions that lead to a transition between stable and chaotic electroconvection modes.

## 1. Introduction

In recent years, the field of electrodialysis (ED) has been extended beyond its typical use in the production of potable water. Some examples of applications of ED that have gained increasing attention are the treatment of industrial wastewaters to recover resources and minimize the discharge of hazardous waste into the environment [1,2], and the separation and upconcentration of products in the biotechnological and food sectors [3,4]. Such progress is being accompanied, not only by the development of new and functional membrane materials [5], but also by exploiting new operating modes in ED [6].

**Figure 1:**
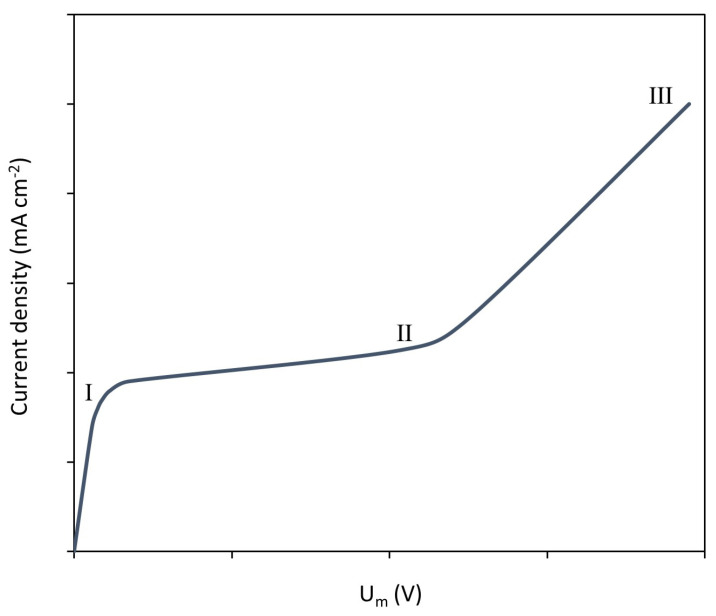
Typical current–voltage curve of a monopolar ion-exchange membrane.

One of the most significant limiting phenomena in ED is concentration polarization, which involves the development of concentration gradients in the solution next to ion-exchange membranes and leads to increased membrane voltage drops. This occurs as a consequence of the selective transport of counter-ions through ion-exchange membranes. Under galvanostatic operation of ED systems, the severity of concentration polarization increases with the level of applied current density. Considering ion transport caused by migration and diffusion, a limiting state is attained when the electrolyte concentration becomes negligible at the membrane surface facing the diluted compartment [7,8]. The current density at which this happens is called the limiting current density, $i_{lim}$, which can be obtained from current–voltage curves. Although some authors have detected more than three regions in current–voltage curves [9], typical curves for monopolar membranes mainly show three regions (see Figure 1):

- Region I (quasi-ohmic): the current density increases linearly with the voltage until $i_{lim}$ is reached. In this region, ion flux is proportional to the applied electric field.
- Region II (plateau): in this region, an increase in the applied voltage implies a subtle or almost absent increase in current density. The scarcity of ions near the diluted membrane surface is the main cause for larger membrane resistances.
- Region III (overlimiting): if the applied voltage is further increased, transport phenomena other than diffusion and migration arise, and current densities above $i_{lim}$ can be registered.

The possibility of increasing the transport of ions for a given membrane surface by operating in the regime of overlimiting currents would facilitate more compact designs, and reduce investment costs and operating times in ED. The most common overlimiting transport mechanisms that can provide an increased ion flux across the membrane are water dissociation and electroconvection [10]. In the case when water dissociation at the membrane predominates, salt ions are not the ones responsible for the increase in current above $i_{lim}$; instead, the generated H${}^{+}$ and OH${}^{-}$ ions induce pH changes in the compartments. Electroconvection was defined by Balster et al. [11] as a nongravitational free convection in the macroscopic area of electrolyte solutions, arising from the interactions of a self-consistent electric field with the corresponding space charge. This type of convection implies an enhanced transfer of salt ions, and is strongly affected by different factors, such as the degree of homogeneity of membrane materials and the presence of surface reliefs [12,13,14], the type of ions involved [15] or the level of applied current density [16,17]. Thus, it is possible to act on ED systems to conveniently promote electroconvection and operate at currents above $i_{lim}$.

When electroconvection emerges, microscopic vortices develop next to the depleting membrane surface and provide an increased mixing with solution layers that are more concentrated; consequently, higher desalination rates can be achieved. Nonetheless, such an advantage becomes possible at the cost of low energy efficiencies, i.e., the voltage drop corresponding to the plateau region is not effectively used to promote ion transport through the membrane [16]. An early onset of electroconvection is key to reduce the specific energy expenditure associated with overlimiting current densities. Thus, different strategies to induce this type of overlimiting mass transfer mechanism have been explored. For instance, it has been proven via numerical simulations that the presence of internal fabrics in membrane structures contributes to stabilize electroconvective vortices in electrodialysis [18,19]. Another factor that can alter the evolution of electroconvective vortices is the presence of a forced convective flow. P. Magnico [20] detected by means of numerical simulations that a forced flow affects the way in which adjacent electroconvective vortices associate and form clusters. Urtenov et al., also found out that stable electroconvection (i.e., electroconvection of the second kind) appeared at homogeneous flat membrane surfaces in the presence of forced flow. This mode of electroconvective transport was previously associated only with the presence of curved or electrically heterogeneous surfaces [21]. Kwak et al. [22] were able to confirm by means of numerical simulations and experiments with an ED cell coupled to a fluorescent microscope that forced flow has a sheltering effect on the electroconvection zone.

The need to develop tools to identify the relative importance of the different parameters affecting electroconvection and their cross-related effects was stressed by Barros et al. [10]. In the present work, chronopotentiometry, an electrochemical characterization technique that is relatively easy to implement in a variety of ED devices, is combined with signal frequency analysis as a tool to identify patterns of electroconvection at various levels of current density. This analysis is implemented using two salt solutions, homogeneous and heterogeneous membranes, and different levels of forced convection.

## 2. Materials and Methods

### 2.1. Membranes and Electrodialysis Cell

A total of two different anion-exchange membranes were investigated in this study. While research on electroconvection is more commonly conducted using cation-exchange membranes, there are few works focusing on anion-exchange membranes. AMV-N (SELEMION), with a structure consisting of a polyolefin polymeric matrix with quaternary amines as fixed ion exchange sites, was selected as the homogeneous membrane. It has an ion exchange capacity of 1.85 meq/g of dry membrane and a typical thickness varying between 80 and 120 μm. HC-A (IONSEP) was the heterogenous anion-exchange membrane selected for this research. It contains fibers to increase its mechanical strength and its fixed ion exchange sites are also quaternary amines. HC-A has an ion exchange capacity of 1.85 meq/g dry membrane and a typical thickness of 420 μm. Nafion 117 (Dupont) was used as the auxiliary cation-exchange membrane.

**Figure A1:**
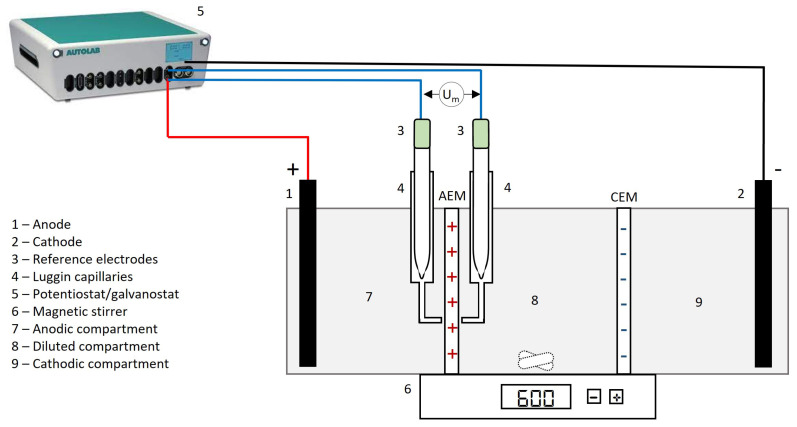
Experimental setup of the three-compartment cell.

The experiments were performed in a three-compartment cell of 125 mL each (anodic, diluted, and cathodic), the setup of the cell is shown in Figure A1, which is described in more detail in previous studies [23,24]. The anodic compartment is separated from the diluted one by the anion-exchange membrane, and the cathodic section is separated from the diluted one by the cation-exchange membrane. The anode and cathode were two graphite bars. Two Ag/AgCl (3M KCl) reference electrodes immersed in Luggin capillaries were placed at both sides of the membrane under study (at 1 mm of the surface) to measure the voltage drop across the membrane system ($U_{m}$). A potentiostat/galvanostat (Autolab PGSTAT 302 N) was used as the power source. The effective membrane area was 1 cm${}^{2}$. Before each experiment, the membranes were immersed in the electrolyte to balance their fixed ion exchange sites during at least 24 h.

During the tests, a magnetic stirrer with speed control (PCE Instruments, PCE-MSR 110) was used in the diluted compartment. The behavior of the system at different stirring rates was analyzed (Table 1). Two different solutions were employed to evaluate differences between the transport of monovalent and divalent anions at a central value of stirring rate: 0.05 M NaCl (NaCl 99.99%, J. T. Baker) and 0.05 M Na${}_{2}$SO${}_{4}$ (Na${}_{2}$SO${}_{4}$ 99%, Panreac Applichem) (Table 2). The electrolyte solutions were prepared with distilled water.

### 2.2. Electrochemical Characterization of the Membrane-Electrolyte Systems

Constant current densities were applied for 300 s; after this time, system relaxation takes place for at least 100 additional seconds, where the concentration profiles at both sides of the membrane become restored. The values of $U_{m}$ were registered every 0.5 s. pH values were also measured after the end of some chronopotentiograms to evaluate the significance of water dissociation in the different current density regimes. All chronopotentiometric experiments were carried out at room temperature (25 °C). The current-voltage curves of both membranes were obtained by plotting the last value of $U_{m}$ registered in each chronopotentiogram versus the applied current density. These curves were used to identify the different mass transfer regimes, and to determine the limiting current density ($i_{lim}$) and the plateau length ($l_{plateau}$) for each membrane-electrolyte combination.

### 2.3. Treatment of Chronopotentiometric Data and Fourier Transform Analysis

The chronopotentiograms obtained at current densities above $i_{lim}$ were further examined by analyzing the shape of the recorded signal fluctuations, since such fluctuations are associated with the development of electroconvective vortices [25,26].

The average amplitude of $U_{m}$ oscillations was extracted from the chronopotentiograms and its evolution at increasing $i/i_{lim}$ values was compared at different stirring rates for the two membranes under study.

**Figure 2:**
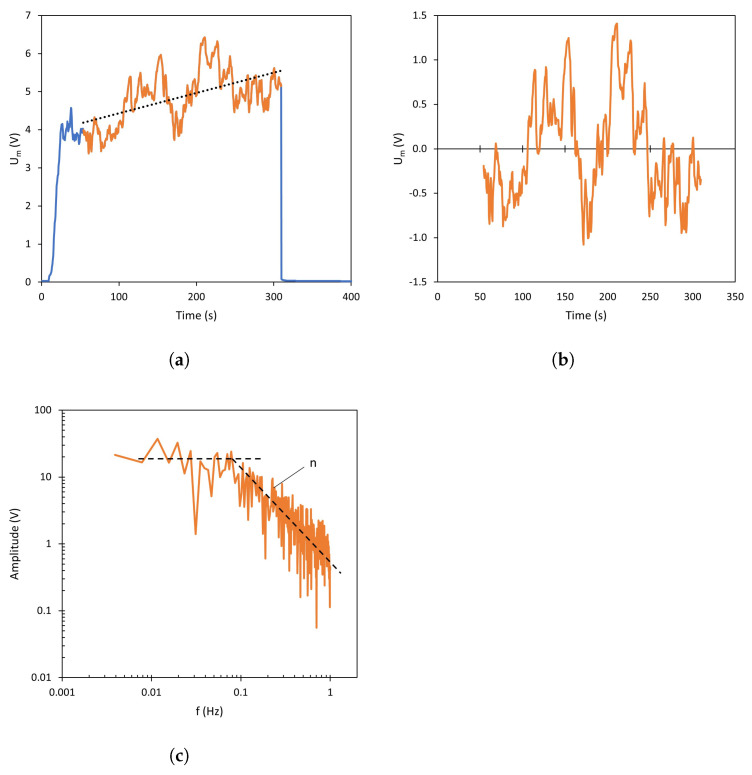
Treatment and standardization of chronopotentiograms for the FFT analysis. (**a**) Original chronopotentiogram before treatment, (**b**) after treatment, and (**c**) resulting spectrum obtained after the signal treatment via Fast Fourier Transform. Dashed lines represent the average trend of the data.

In addition, the oscillatory behavior of $U_{m}$ was analyzed in detail by computing the Fast Fourier Transform (FFT) of the signal. Before performing the frequency analysis, it is necessary to normalize the curves obtained for each current density in order to remove the drift and the background quasi-ohmic component of the signal (see Figure 2a,b). After that, the FFT is calculated (Figure 2c).

The frequency of the signal, *f* (Hz), has been obtained using Equation (1).

(1) $$f=\frac{k}{N\times t_{sampling}}$$

where *k* is a data counter; *N* is the number of values used for the frequency analysis, and $t_{sampling}$ is the interval between samples, 0.5 s in this study. The calculated FFT (Equation (2)) is represented in absolute value as a function of the frequency in logarithmic plots, obtaining Fourier spectra, such as that shown in Figure 2c.

(2) $$FFT\left(k\right)=\sum_{k=0}^{N-1}U_{m}\left(k\right)\times e^{-\left(\frac{i\times 2\pi \times k}{N}\right)}\;\;k=0,1,\ldots ,N-1$$

In Equation (2), $i=\sqrt{-1}$.

As explained below in Section 3, depending on the specific operating conditions, these spectra may have an almost constant slope or adopt two different slopes determined by the signal frequency: in the example shown in Figure 2c, the spectrum is almost flat at low frequencies; while at high frequencies, a negative slope can be observed.This change in the shape of the spectrum indicates an increase in the amplitude of the medium-frequency components of the signal [27]. The slope of the high-frequency regions (coefficent *n*) was extracted as a characteristic parameter related to the progress of electroconvection in the different systems.

## 3. Results and Discussion

### 3.1. Effect of Forced Convection, Applied Current Density and Membrane Type on the Current–Voltage Characteristics: $i_{lim}$ and $l_{plateau}$

**Figure 3:**
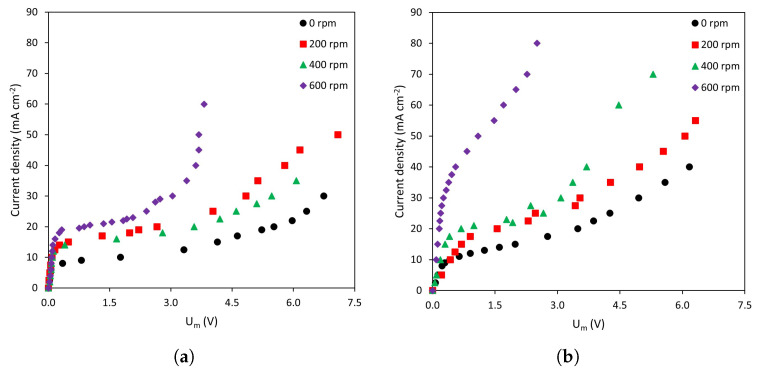
Current–voltage curves obtained at various stirring rates. (**a**) Homogeneous membrane, AMV-N; (**b**) Heterogeneous membrane, HC-A.

Prior to analyzing the dynamics of transmembrane voltage drops, general trends observed in the current–voltage curves of the different membrane–solution systems will be analyzed. Figure 3 shows the current–voltage curves obtained in NaCl solutions with the (a) homogeneous AMV-N and the (b) heterogeneous HC-A anion-exchange membrane at various stirring rates. The three characteristic regions of current–voltage curves described previously can be observed in all systems regardless of the operating conditions. However, the shape of the curves depends on both the membrane type and the intensity of forced convection.

Regarding the effect of stirring rate on the shape of the curves, it can be observed that, for a given current density, the values of $U_{m}$ become smaller as the stirring rate increases. This represents a decrease in membrane resistance as a consequence of the intensified forced convection, which contributes to the thinning of the diffusion boundary layers. As expected, the differences caused by stirring are less evident in the quasi-ohmic region, because for $i<i_{lim}$ concentration profiles are not fully developed. However, at values approaching $i_{lim}$, the mixing effect can already be seen. An increase in the stirring rate also causes an increase in the $i_{lim}$ values and the initiation of overlimiting currents for lower values of $U_{m}$. These trends can be observed in both types of membranes.

**Figure 4:**
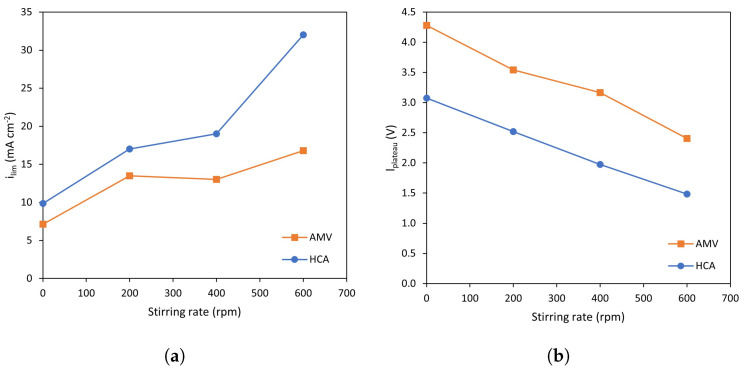
Comparison between the characteristic parameters at various stirring rates for both membranes: (**a**) Limiting current density, and (**b**) plateau length.

As for the current–voltage characteristics, the values of $i_{lim}$ and $l_{plateau}$ are represented in Figure 4. It can be confirmed from these plots that the effect of stirring is analogous for both membranes: at increasing stirring rates, $i_{lim}$ values increase (Figure 4a) and the plateau length decreases (Figure 4b). In previous works, it is often presented that heterogeneous membranes exhibit lower $i_{lim}$ values than homogeneous ones due to their lower fraction of conducting regions [28]. However, the trend is reversed in the present work, which may be explained by a lower permselectivity of the heterogeneous HC-A membrane. Choi et al., also found a change in the sequence of $i_{lim}$ values in a study involving three membranes of differing degrees of heterogeneity [29]. Zabolotosky et al., compared the behavior of homogeneous and heterogenous membranes observing a similar trend: larger values of limiting current density were obtained with heterogeneous membranes [30].

Regarding the plateau length, as commented above, forced convection favors the registration of overlimiting currents at lower potential values, which is confirmed by the decreasing $l_{plateau}$ values at higher stirring rates (Figure 4b). This parameter is lower for the heterogeneous membrane, HC-A. In this case, the trend coincides with that commonly reported in previous works: membrane surface reliefs and non-conducting parts cause an earlier onset of electroconvection [31,32].

**Figure 5:**
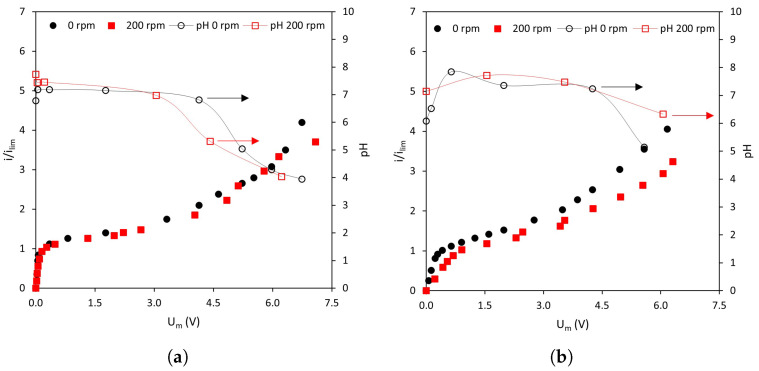
Current–voltage curves obtained at 0 and 200 rpm and pH evolution of the solution in the diluted ED compartment vs. $U_{m}$. (**a**) AMV-N, (**b**) HC-A.

Figure 5 shows the pH evolution in the diluted compartment during the registration of current–voltage curves. It can be confirmed that electroconvection is the principal overlimiting mechanism for the range of currents between $1<i/i_{lim}<2$, since pH values remain constant within this range. However, above 2 $\times \;i_{lim}$, water dissociation also becomes a significant contribution to ion current as denoted by the decrease in pH for both membrane systems. Upon the depletion of ions near the diluted membrane surface, water molecules become highly polarized and give rise to H${}^{+}$ and OH${}^{-}$ ions, remaining the former in the diluted compartment and migrating the latter through the respective anion-exchange membrane. It is interesting to note that the range of currents at which changes in pH were noticed are similar for both membranes and at different degrees of stirring. In previous works, a similar evolution of pH was registered, but changes in pH started from current densities near $i_{lim}$ [33,34].

### 3.2. Amplitude of Membrane Voltage Oscillations

**Figure 6:**
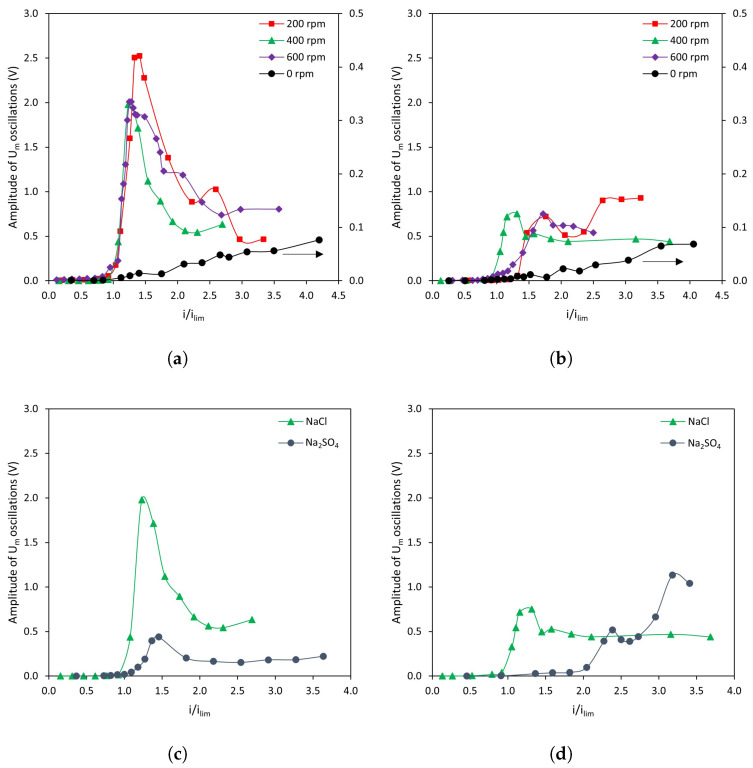
Evolution of the amplitude of membrane voltage oscillations at various levels of stirring rate. (**a**) AMV-N with NaCl as electrolyte, (**b**) HC-A with NaCl as electrolyte. Evolution of the amplitude of membrane voltage oscillations with NaCl and Na${}_{2}$SO${}_{4}$ at 400 rpm. (**c**) AMV-N, (**d**) HC-A.

Figure 6a,b show the amplitude of membrane voltage oscillations registered for different stirring rates as a function of *i*/$i_{lim}$. Overall, the evolution follows two differentiated trends: in systems without forced convection (0 rpm, values read in the right axis), the amplitude increases almost linearly with the applied current density; whereas under the application of stirring, the amplitude increases sharply after surpassing $i_{lim}$, and then decreases and becomes stable.

Under the absence of stirring, the increase in amplitude is almost proportional to the increase in current density for both membranes. This evolution of membrane voltage drop can be ascribed to the gradual growth of electroconvection vortices as the electric field is intensified. Rubinstein et al., were able to visualize electroconvective vortices in the absence of forced flow and determined their height, from which they could confirm a linear relationship between the vortex size and the applied voltage [35].

For stirred systems, a sharp increase in voltage oscillations is detected when $i_{lim}$ is exceeded. Krol et al. [25] also detected an increase in the amplitude of membrane voltage oscillations at increasing applied current densities in systems with forced convection. Similarly as for 0 rpm, an intensified electric field involves the formation of bigger electroconvective vortices. However, it must be noted that the amplitude of voltage oscillations is significantly larger in the case of forced convection. Such a substantial difference may be explained by the thinning of the diffusion boundary layers as a consequence of an improved fluid mixing, which implies that the solution that comes from the bulk and reaches the outer edge of the vortices is much more concentrated as compared to the unstirred system. Consequently, the peak-to-peak voltage signal corresponds to larger concentration differences. The maximum in amplitude of $U_{m}$ oscillations agrees well with the conclusions of a previous study, where it was confirmed that a pressure-driven flow can confine the region where electroconvection develops as long as the vortices do not become chaotic [22]. Kwak et al., reported that the height of stable electroconvection vortices is proportional to the square of applied voltage when shear-flow is imposed [36]. If the electric field is further intensified, vortices destabilize and turn chaotic, thus being swept by the forced flow [37]. This sweeping effect agrees well with the observed decrease in the signal amplitude for $i/i_{lim}>1.75$.

The maximum amplitude of $U_{m}$ oscillations reached with the homogeneous AMV-N membrane is 2.5-fold that achieved with the heterogeneous HC-A one. This difference could be caused by the presence of reliefs in the surface of the HC-A membrane, as a consequence of the fiber cloth used to increase its mechanical resistance. Such undulations favor an earlier transition to unstable electroconvection modes, hence limiting the maximum value reached in $U_{m}$ amplitude.

Figure 6c,d show a comparison between the amplitude of membrane voltage oscillations when using different counter-ions (i.e., monovalent and multivalent anions). In both cases the trends observed with Na${}_{2}$SO${}_{4}$ are similar to those observed with NaCl. With the homogeneous membrane, AMV-N, the amplitude reaches a maximum and then stabilizes in a lower value (Figure 6c). However, the amplitude of the oscillations is significantly smaller for Na${}_{2}$SO${}_{4}$. The influence of the electrolyte is notorious using the HC-A heterogeneous membrane (Figure 6d). With Na${}_{2}$SO${}_{4}$, the amplitude of membrane voltage oscillations increases when current density values larger than 2 $\times \;i_{lim}$ are applied. The conductivity of SO${}_{4}^{2-}$ ions is 160 × 10${}^{-4}$ m${}^{2}$ S mol${}^{-1}$, higher than the value for Cl${}^{-}$ ions (76.31 × 10${}^{-4}$ m${}^{2}$ S mol${}^{-1}$) [38]. It is possible that the generation of electroconvective vortices involving the movement of ions of higher conductivity makes the changes in transmembrane voltage drop less notorious, even when larger current densities are applied. In previous studies, it was already observed that lower mobility of the ion provides a more appreciable amplitude of $U_{m}$ oscillations [39]. Here, it is interesting to note that, in a previous work involving cation-exchange membranes, larger amplitudes in voltage oscillations were also related to the degree of hydration of the counterion [40]; however, in the present work, the role of conductivity seems to be more important: the larger hydrated radius for sulfate ions does not correspond to larger oscillation amplitudes in comparison with those registered with chloride ions.

### 3.3. FT Analysis of Membrane Voltage Signals

**Figure 7:**
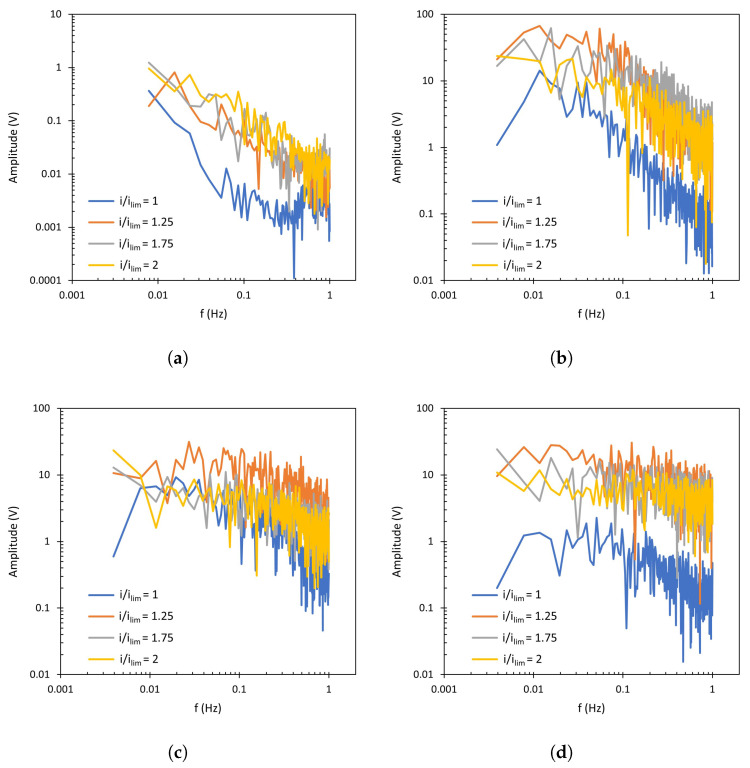
Fourier Transform of the membrane AMV-N at (**a**) 0 rpm, (**b**) 200 rpm, (**c**) 400 rpm, (**d**) 600 rpm.

The FFT spectra obtained from the chronopotentiograms conducted with NaCl solutions and the AMV-N membrane are shown in Figure 7, where the amplitude of each component frequency of the $U_{m}$ signal is represented. The original chronopotentiometric curves for this membrane are shown in the Appendix A (Figure A1). The most remarkable change between different spectra is that related to the presence of forced convection. For the unstirred system (Figure 7a), an approximately linear decrease in amplitude of the signal components can be observed at increasing frequencies, with this trend being common to all levels of current density. In this system, a notable increase in signal amplitude can also be seen at $i/i_{lim}>1$, especially at frequencies around 0.1 Hz. The formation of vortices due to electroconvection when the applied electric field is intensified can thus be related to this frequency domain. When the ED compartment is stirred (Figure 7b–d), the FFT spectra show two well-differentiated regions: one that is almost flat at frequencies lower than 0.1 Hz, and a second one with decreasing amplitudes at frequencies higher than 0.1 Hz. Here, it is remarkable that the increase in amplitude of the flat region (*f* < 0.1 Hz) is already observed at $i/i_{lim}=1$, so that it can be mainly attributed to stirring. In some cases, the signals at 200 and 400 rpm are slightly higher than the signal obtained at 600 rpm, which could be caused by the sweeping effect of vortices at the highest level of forced convection. In general, the strongest interaction between high levels of forced convection and current density is observed at 0.1 Hz, where the signal amplitude increases the most and causes a transition between the flat and the descending regions of the FFT spectra.

**Figure 8:**
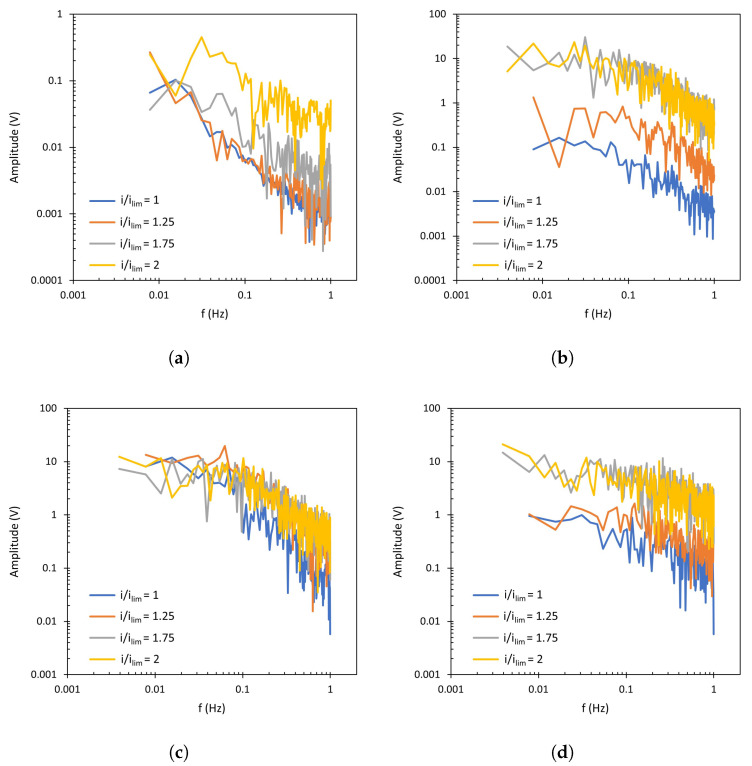
Fourier Transform of the membrane HC-A at (**a**) 0 rpm, (**b**) 200 rpm, (**c**) 400 rpm, (**d**) 600 rpm.

**Figure A2:**
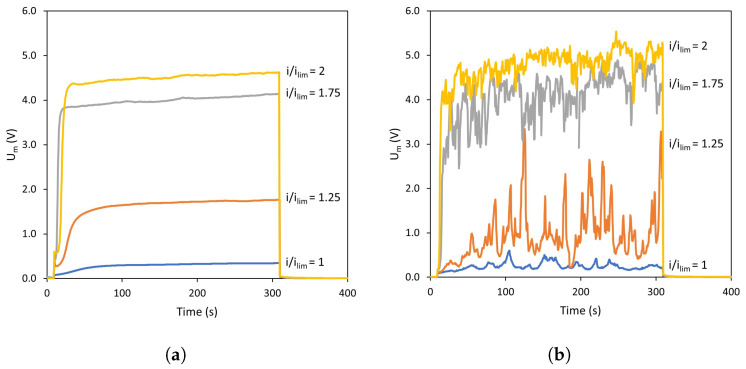

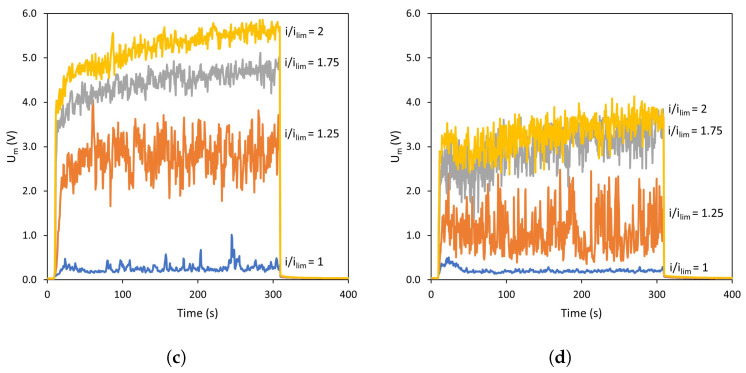
Chronopotentiometric response obtained with AMV-N membrane and NaCl as electrolyte at (**a**) 0 rpm, (**b**) 200 rpm, (**c**) 400 rpm, (**d**) 600 rpm.

The spectra obtained with the heterogeneous HC-A membrane are presented in Figure 8. These correspond to the chronopotentiograms shown in Figure A2 of the Appendix A. In general, the trends observed for both membranes are similar. At 0 rpm, for all applied values of *i*/$i_{lim}$, the signal amplitude decreases linearly at increasing frequencies. When the applied current density exceeds $i_{lim}$, the generation of electroconvective vortices also increases the amplitude of the signal. For the stirred systems, the notorious increase in amplitude at around 0.1 Hz causes the transition between the two characteristic regions, described above for the AMV-N membrane and also observed previously by other researchers [41]. Under stirring conditions, the amplitude value is at least one order of magnitude higher than that obtained without stirring.

**Figure 9:**
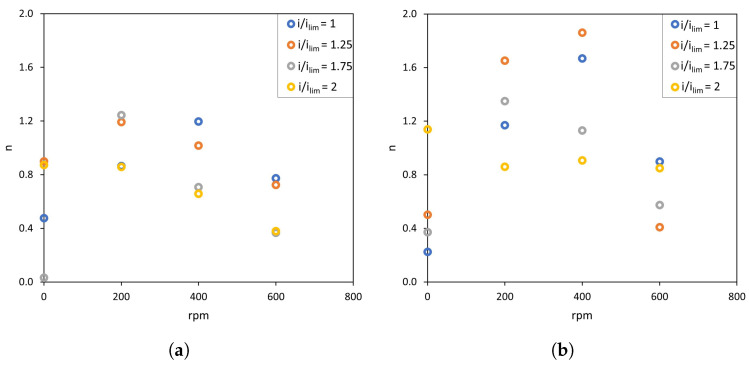
Evolution of the *n* value at various stirring rates. (**a**) AMV-N, (**b**) HC-A.

Regarding the effect of different levels of stirring rates, it is difficult to identify differences from the complete FFT spectra. However, these differences can be better quantified by the comparison of *n* values, that is, the slope of the spectrum registered at frequencies higher than 0.1 Hz which exhibit a descending trend. This parameter reflects the interactions between forced convection caused by stirring and electroconvection caused by intensified electric fields. In Figure 7 and Figure 8, enhanced electroconvection as a consequence of vortex sheltering by forced flow implies an increase in the signal components registered at around 0.1 Hz, this being translated into higher *n* values. The values of the slope (*n*) of Fourier transforms are summarized in Figure 9. In both membranes, for values of $i/i_{lim}<2$, a maximum value of *n* is obtained at 200 or 400 rpm (depending on the system), coinciding with the conditions at which forced convection confines electroconvective vortices. As compared to the conditions without stirring, the thinning of the diffusion boundary layer and the growth of the electroconvection zone takes place simultaneously, and this is translated into an overlapping between both phenomena. Liu et al. also observed via numerical simulations that increasing shear flow velocity can shelter chaotic electroconvection, while increasing the voltage increases it [42]. However, increasing the stirring rate up to 600 rpm implies a further decrease in *n*, demonstrating that vortices can be swept by the fluid flow. Li et al. also observed a suppression of electroconvective and morphological instabilities by an imposed flow of the electrolyte [37]. Operating at 2 $\times \;i_{lim}$, the maximum in *n* is not observed. Instead, the values of n decrease at increasing stirring rates. At such a high value of current density, electroconvection evolves into an unstable mode, where the formation of chaotic vortices occurs, and the sweeping effect of forced convection predominates. Here, in addition to the sweeping effect, dissociation of water at the membrane surface may also distort the formation of vortices.

**Figure 10:**
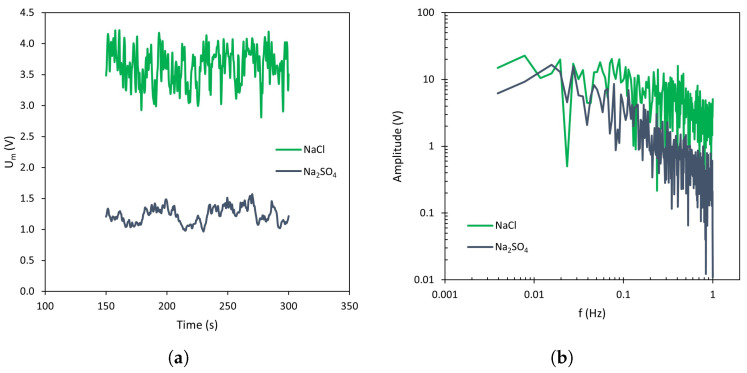

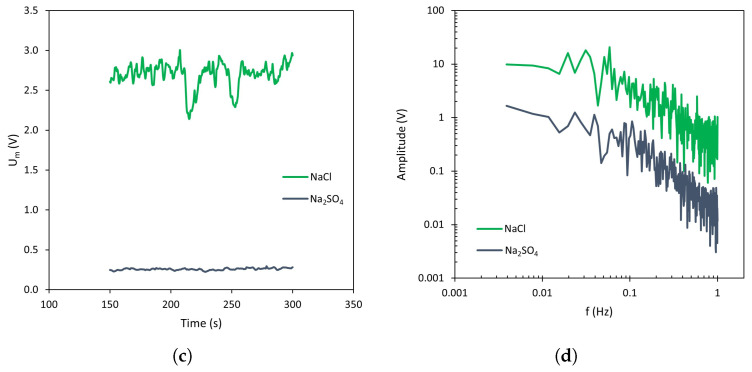
(**a**) Chronopotentiometric response obtained with AMV-N membrane at 1.5 $\times \;i_{lim}$ at 400 rpm, (**b**) Fourier Transform, (**c**) Chronopotentiometric response obtained with HC-A membrane at 1.5 $\times \;i_{lim}$ at 400 rpm, (**d**) Fourier Transform.

In Section 3.2, the effect of the counter-ion type on the amplitude of membrane voltage oscillations was analyzed, confirming the influence of the anion on the membrane behavior. When membrane voltage signals are studied by Fourier Transform, a different response is also detected for both types of counter-ions (Figure 10). The spectra obtained with the homogeneous membrane (Figure 10b), corresponding to the curves shown in Figure 10a, show a similar behavior in the regime of low-frequency values, where the effect of forced convection on the oscillatory signal is dominant. However, in the high-frequency region (where the contribution of electroconvection to the signal is more important) larger amplitudes are registered with the monovalent anion. With the heterogeneous membrane (Figure 10c,d), the difference between both electrolytes is even more relevant, since higher amplitude values are obtained with NaCl across the whole frequency range. As commented in the previous section, this behavior may be associated with the lower conductivity of Cl${}^{-}$ as compared with SO${}_{4}^{2-}$ ions. Changes associated with a given concentration difference measured by the reference electrodes become more significant for species with a lower conductivity.

## 4. Conclusions

This work establishes a novel method combining chronopotentiometry and Fourier transform analysis to investigate the oscillatory behavior of transmembrane voltage signals associated with electroconvection. The influence of different parameters on the membrane response, such as the heterogeneity of the membranes, the intensity of forced convection, the level of applied current density and the type of counter-ion, has been analyzed.

Regarding the amplitude of membrane voltage oscillations, in systems without forced convection, the amplitude increases almost linearly with the applied current density, which is attributed to a gradual increase in the vortices’ size. In stirred systems, the amplitude reaches a maximum value significantly higher than that reached without stirring, thus confirming an important interaction between forced convection and electroconvection. However, at the highest values of current density, the nature of the vortices becomes chaotic and the electroconvection zone overlaps with the stirred region. This results in a sweeping effect of the vortices and a concomitant decrease in the signal amplitude.

Analyzing the FFT spectra in systems without forced convection, the spectra present a single region of decreasing amplitudes at higher frequencies. On the contrary, in systems with stirring, the FFT spectra clearly show two distinct zones, a flat one at frequencies lower than 0.1 Hz, that is mainly related to forced convection; and a descending one at frequencies above 0.1 Hz, that is strongly influenced by the development of electroconvection. In conclusion, the FFT analysis of the $U_{m}$ amplitude reveals that the effect of electroconvection on the signal is especially relevant at a characteristic frequency of 0.1 Hz. The influence of different levels of stirring rates has been studied with the slope values of the descending region (*n*). Higher values of this coefficient correspond to enhanced electroconvection due to the sheltering of vortices by the forced flow. In general, maximum values are obtained at applied stirring rates lower than 600 rpm and current densities below 2 $\times \;i_{lim}$.

## Figures and Tables

**Table 1:** Experimental conditions applied in the electrochemical characterization of the membrane-electrolyte systems.

Membrane	Electrolyte	Stirring Rate	Reynolds Number
AMV-N	NaCl	0 rpm	0
AMV-N	NaCl	200 rpm	1498
AMV-N	NaCl	400 rpm	2995
AMV-N	NaCl	600 rpm	4493
HC-A	NaCl	0 rpm	0
HC-A	NaCl	200 rpm	1498
HC-A	NaCl	400 rpm	2995
HC-A	NaCl	600 rpm	4493

**Table 2:** Experimental conditions applied in the evaluation of the effect of the type of counter-ion on the development of electroconvection.

Membrane	Electrolyte	Stirring Rate	Reynolds Number
AMV-N	NaCl	400 rpm	2995
AMV-N	Na${}_{2}$SO${}_{4}$	400 rpm	2971
HC-A	NaCl	400 rpm	2995
HC-A	Na${}_{2}$SO${}_{4}$	400 rpm	2971

**Figure A3:**
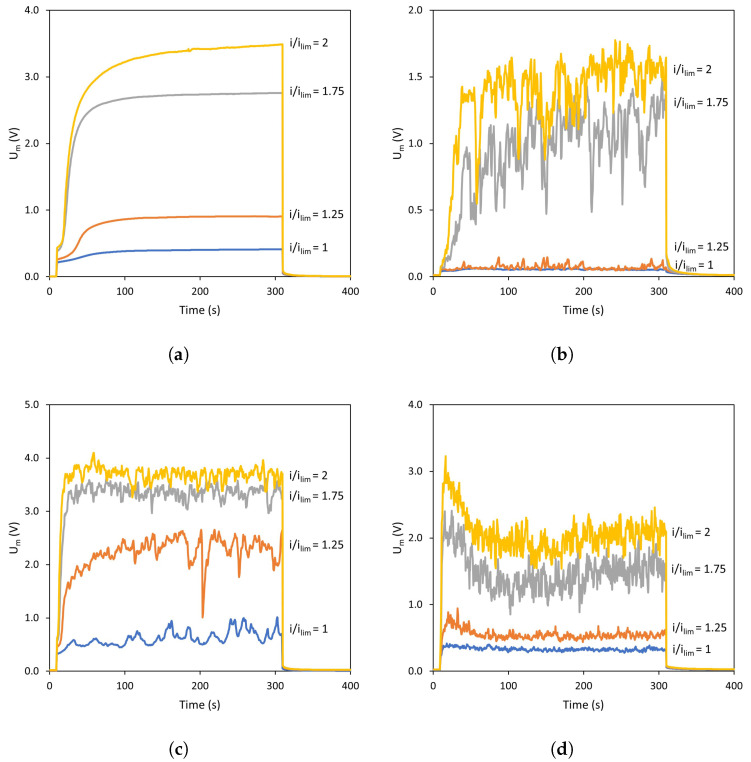
Chronopotentiometric response obtained with HC-A membrane and NaCl as electrolyte at (**a**) 0 rpm, (**b**) 200 rpm, (**c**) 400 rpm, (**d**) 600 rpm.

## Data Availability

The data presented in this study are available on request from the corresponding author. The data are not publicly available due to privacy restrictions.

## Abbreviations

The following abbreviations are used in this manuscript:

ED	Electrodialysis
FT	Fourier Transform
FFT	Fast Fourier Transform
